# Treatment and clinical outcome of clinical T4 esophageal cancer: A systematic review

**DOI:** 10.1002/ags3.12222

**Published:** 2018-12-13

**Authors:** Tomoki Makino, Makoto Yamasaki, Koji Tanaka, Yasuhiro Miyazaki, Tsuyoshi Takahashi, Yukinori Kurokawa, Masaaki Motoori, Yutaka Kimura, Kiyokazu Nakajima, Masaki Mori, Yuichiro Doki

**Affiliations:** ^1^ Department of Gastroenterological Surgery Graduate School of Medicine Osaka University Osaka Japan; ^2^ Department of Surgery Osaka General Medical Center Osaka Japan; ^3^ Department of Surgery Faculty of Medicine Kindai University Osaka Japan

**Keywords:** conversion surgery, definitive chemoradiation, esophageal cancer, induction chemotherapy, T4

## Abstract

**Background:**

Survival of patients with cT4 esophageal cancer is dismal. Although the optimal treatment strategy remains to be established, two treatment options are available for cT4 esophageal cancers: definitive chemoradiation (dCRT) and induction treatment followed by conversion surgery (CS). However, little is known concerning the differences in clinical outcome between patients with T4 esophageal tumors treated with dCRT and those eventually treated with CS.

**Methods:**

A systematic search of the scientific literature on PubMed/MEDLINE was carried out using the keywords “T4 esophageal cancer,” “invading (involving) adjacent organ,” “definitive chemoradiation,” “induction therapy,” “salvage surgery,” and “conversion surgery,” obtaining 28 reports published up to July 2018.

**Results/Conclusion:**

We found that CS was superior to dCRT with respect to local disease control and short‐term survival; however, CS was associated with relatively higher perioperative mortality and morbidity. Alternatively, although dCRT might often cause fistula formation, a clinical complete response to dCRT is likely to lead to a better prognosis. Recent advances in chemotherapeutic agents have led to triple induction chemotherapy, with docetaxel, cisplatin, and 5‐fluorouracil (DCF), which has shown promise as an initial induction treatment for cT4 esophageal cancer. Indeed, this regimen could control both local and systemic disease, which enables curative resection without preoperative CRT. Moreover, some appropriate changes in perioperative management and intensive systemic chemotherapy might enhance patient outcome. Randomized controlled trials with a large sample size are needed to establish the standard treatment for cT4 esophageal cancer.

## 1 INTRODUCTION

Esophageal cancers tend to invade adjacent organs, including the trachea, bronchus, lung, and aorta, as a result of the lack of serosa in the esophagus and the fact that this conduit is located in a very narrow mediastinal space.^1^, ^2^ Tumors that invade adjacent organs are classified as T4, according to the TNM staging system of the International Union against Cancer (UICC). Despite recent advances in multidisciplinary treatments, the prognosis of patients with T4 esophageal cancer remains unsatisfactory.^3^ Although esophageal cancer is associated with a high incidence of morbidity and mortality, treating with surgery alone, where neighboring organs are resected together with an esophagectomy, has not improved survival.^4^, ^5^, ^6^, ^7^ Similarly, definitive chemoradiation (dCRT), a maximum‐dose irradiation together with chemotherapy used as a curative treatment which many investigators consider the most suitable treatment for T4 esophageal cancer, has not dramatically contributed to improving patient survival.^8^ As a result of a paucity of evidence, a treatment strategy for T4 esophageal cancer has not been established to date. According to the Guidelines for Diagnosis and Treatment of Carcinoma of the Esophagus, 2017, the current standard chemotherapeutic regimen for treating esophageal cancer is 5‐fluorouracil (5‐FU) combined with cisplatin^9^ (CF) because of their synergistic radiosensitizing effects.^10^ Previous studies have reported that concurrent CRT with a CF regimen was effective for treating advanced esophageal cancers, including T4 tumors.^1^, ^11^ Thus, two modalities are currently in use for the treatment of cT4 esophageal tumors:^12^, ^13^ dCRT^14^, ^15^, ^16^, ^17^, ^18^, ^19^ and induction chemotherapy or CRT, followed by conversion surgery (CS).^12^, ^13^, ^20^, ^21^, ^22^, ^23^, ^24^, ^25^, ^26^, ^27^, ^28^, ^29^ Theoretically, when surgery completely removes the tumor, survival should be prolonged, regardless of the T stage. Thus, effective induction treatments must be established for T4 tumors to achieve curative resections, even for initially unresectable tumors.^30^ However, to our knowledge, there is little or no information on the differences in clinical outcome between patients with T4 esophageal tumors treated with dCRT and those eventually treated with CS.

Recently, new triplet chemotherapy regimens have been reported to yield high response rates for esophageal squamous cell carcinoma (ESCC).^31^, ^32^ In particular, docetaxel plus CF (DCF) was shown to be more effective for treating ESCC than the standard treatment of CF or CF plus adriamycin (ACF).^31^ Some studies showed promising results when induction chemotherapy with the DCF regimen was applied before carrying out CS for cT4 ESCC.^20^, ^24^, ^30^ In the present review, we focus on these treatments and the outcomes in patients with T4 esophageal cancer, and we discuss future perspectives regarding these modalities.

## 2 MATERIALS AND METHODS

We conducted a systematic search of the scientific literature on PubMed/MEDLINE to obtain all relevant articles involving T4 esophageal cancers published up to July 2018. In the searches, we excluded all non‐English articles. To avoid duplications of data, articles from the same unit or hospital were included only once, when data were being updated in a later publication. The search terms were “T4 esophageal cancer,” “invading (involving) adjacent organ,” “definitive chemoradiation,” “induction therapy,” “salvage surgery,” and “conversion surgery.” All available major publications (primarily from high‐volume surgical centers) were considered. Articles were selected when the abstract indicated that data were collected on patients with T4 esophageal cancer included in randomized controlled trials (RCT), other cohorts, or comparative studies. We also reviewed the reference lists of these articles to find additional candidate studies. For the present study, data were taken from the published reports; authors were not contacted to obtain additional information. Therefore, articles that lacked necessary data, including survival information according to each treatment group, were excluded from this systematic review. Reports with fewer than 10 cases were also excluded from this study.

## 3 RESULTS

### 3.1 Studies included in the present review

A fiow chart of the article selection process is shown in Figure S1. A total of 28 articles regarding dCRT or/and induction treatment, followed by CS for cT4 esophageal cancer were finally selected (Table 1).

**Table 1:** Summary of definitive chemoradiotherapy and conversion surgery for patients with clinical T4 esophageal cancer

Authors	Design	Year	N	Histology (SCC/AC/Other)	Treatment (N)		Total radiation dose/chemotherapy regimen		1/3/5‐year overall survival rate (%)	
					CRT	CS	CRT	CS	CRT	CS
Yokota et al^20^ (COSMOS)	P2	2016	48	47/0/1	–	20	–	DCF (n = 18) DCF+ 30‐60 Gy/CF (n = 2)	–	100/90/NA
Ohira et al^41^	Retro	2015	91	91/0/0	–	40	–	40‐60 Gy/CF or FN	–	NA/NA/51
Akutsu et al^21^	Retro	2014	40	40/0/0	–	28 (early responders) 12 (late responders)	–	40 Gy/CF 53 Gy/CF	–	74/48/26 72/36/37
Shimoji et al^22^	Cohort	2013	43	42/1/0	–	30	–	40‐66 Gy/FN (n = 17) FAN (n‐26)	–	52/35/35
Pimiento et al^23^	Retro	2013	45	6/36/3	–	45	–	NA/platinum‐based	–	75/50/35
Miyata et al^24^	Retro	2012	169	169/0/0		98		40‐60 Gy/CF (n = 41) FAP or DCF (n = 41) both (n = 16)	–	NA/48/40
Miyoshi et al^26^	Retro	2009	42	42/0/0	–	42	–	40 Gy/CF or ACF	–	66/45/38
Seto et al^12^	Retro	2007	88	88/0/0	29	59	60 Gy/CF	40 Gy/CF	35/7/7	68/38/20
de Manzoni et al^25^	Retro	2007	51	51/0/0	–	51	–	50‐60 Gy/CF	–	NA/9/6
Fujita et al^13^	Cohort	2005	53	53/0/0	23	30	60 Gy/CF	36 Gy (+24 Gy^a^)/CF	44/13/13	73/28/17
Noguchi et al^27^	Retro	2003	41	41/0/0	–	41	–	40 Gy/CF	–	24/5/0
Ikeda et al^28^	P2	2001	37	37/0/0	–	37	–	60 Gy/CF	–	45/23/23
Yano et al^29^	Retro	1999	45	45/0/0	–	45	–	40 Gy/CF	–	48/35/25
Van Raemdonck et al^42^	Retro	1997	18	15/3/0		18		36‐50 Gy/CF or MF		62/43/NA
Satake et al^33^	P1/2	2016	33^b^	33/0/0	33	–	DCF→60 Gy/CF	–	79/40/NA	–
Jingu et al^34^	Retro	2016	70	70/0/0	70	–	50‐70 Gy/CF or DCF or FN	–	33 (2y)/24 (4y)	–
Li et al^40^	Retro	2016	56	56/0/0	56	–	54‐60 Gy/CF or DC or PC	–	39/21/NA	–
Miyazaki et al^35^	P1/2	2015	37^b^	36/1/0	37	–	60 Gy/DCF	–	78/44/NA	–
Shinoda et al^36^ (JCOG0303)	rP2	2015	142^b^	142/0/0	71 71	–	60 Gy/low‐dose CF 60 Gy/standard‐dose CF	–	56^b^/26^b^/NA 56^b^/26^b^/NA	–
Higuchi et al^37^ (KDOG0501‐P2)	P2	2014	42^b^	42/0/0	42	–	50.4‐61.2 Gy/DCF	–	66^b^/44^b^/NA	–
Font et al^14^	Retro	2007	19	NA/NA/NA	19	–	66 Gy/docetaxel	–	26/0/0	–
Ishida et al^38^ (JCOG9516)	P2	2004	60^b^	60/0/0	60^b^	–	60 Gy/CF	–	38^b^/23^b^/NA	–
Crosby et al^15^	Retro	2004	27	NA/NA/NA	27	–	50 Gy/CF	–	45/23/NA	–
Kaneko et al^16^	Retro	2003	35	35/0/0	35	–	60 Gy/CF	–	45/8/NA	–
Nishimura et al^19^	P1/2	2002	28	28/0/0	23	–	60 Gy/CF	–	30/NA/NA	–
Itoh et al^17^	Retro	2001	35	33/1/1	35	–	60 Gy/CF	–	38/10/10	–
Ohtsu et al^18^	P2	1999	36	36/0/0	36	–	60 Gy/CF	–	41/14/14	–
Ohtsu et al^39^	Pilot	1995	20^b^	20/0/0	20	–	60 Gy/CF	–	NA	–

5FU, 5‐fluorouracil; AC, adenocarcinoma; CF, 5‐fluorouracil and cisplatin; cohort, cohort study; CRT, chemoradiation; CS, conversion surgery; DC, docetaxel and cisplatin; DCF, docetaxel, cisplatin, and 5‐fluorouracil; FAN, 5‐fluorouracil, adriamycin, and nedaplatin; FN, 5‐fluorouracil and nedaplatin; MF, mitomycin C and 5‐fluorouracil; NA, data not available; P(1/)2, phase (I/)II study, rP2 randomized phase II study; PC, paclitaxel and carboplatin; pilot, pilot study; retro, retrospective study; SCC, squamous cell carcinoma.

^a^postoperative dose. ^b^T4/M1 lym tumors.

### 3.2 Definitive chemoradiation

#### 3.2.1 Chemoradiation regimen

As summarized in Tables 1 and 2, a total of 16 studies^12^, ^13^, ^14^, ^15^, ^16^, ^17^, ^18^, ^19^, ^33^, ^34^, ^35^, ^36^, ^37^, ^38^, ^39^, ^40^ examined the outcome of patients with T4 esophageal cancer after dCRT. Two major clinical studies on dCRT for esophageal cancer conducted in Japan, termed JCOG9516^38^ and JCOG0303,^36^ were carried out primarily by the Japanese Clinical Oncology Group. Of the 16 studies, 13^12^, ^13^, ^15^, ^16^, ^17^, ^18^, ^19^, ^33^, ^34^, ^35^, ^36^, ^38^, ^39^, ^40^ used CF, and three recent studies^34^, ^35^, ^37^ reported the concurrent use of triplet chemotherapy (DCF). Font et al^14^ used a weekly docetaxel regimen (20 mg/m^2^). Concurrent radiotherapy was applied in all studies, with a total external radiation dose of 50‐66 Gy.

**Table 2:** Summary of outcomes in definitive chemoradiotherapy group

Authors	Year	N	Grade 3/4 toxicities (%)		Fistula formation (%)	Mortality (%)	Response rate (%)	cCR rate (%)	1/3/5‐year overall survival rate (%)	
			Acute	Late					cCR	Non‐cCR
Satake et al^33^	2016	33^c^	24^c^ (leukocytopenia) 18^c^ (neutropenia) 15^c^ (dysphagia)	NA	6^c^	0^c^	73	39^c^	NA	NA
Jingu et al^34^	2016	70	44^c^ (leukocytopenia) 17^c^ (esophagitis)	3^c^ (pneumonitis)	5^c^	7^c^	NA	NA	NA	NA
Li et al^40^	2016	56	25 (leukocytopenia)	NA	11	9	61	23	92/55/NA	NA
Miyazaki et al^35^	2015	37^a^	92^c^ (leukocytopenia) 76^c^ (neutropenia) 32^c^ (nausea)	NA	5	0	86	48	NA	NA
Shinoda et al^36^ (JCOG0303)	2015	71^c^ (LDCF) 71^c^ (SDCF)	29^c^ (dysphagia/esophagitis) 21^c^ (leukocytopenia) 21^c^ (anorexia) 26^c^ (leukocytopenia) 23^c^ (dysphagia/esophagitis) 20^c^ (anorexia)	22^c^ (fistula) 18^c^ (dyspnea) 10^c^ (dysphagia) 18^c^ (fistula) 14^c^ (dyspnea) 11^c^ (dysphagia)	22^c^ 18^c^	1^c^ 3^c^	NA NA	1^c^ 0^c^	NA NA	NA NA
Higuchi et al^37^ (KDOG0501‐P2)	2014	42^c^	71^c^ (leukocytopenia) 57^c^ (neutropenia) 38^c^ (febrile neutropenia)	8^c^ (esophagitis /stenosis/fistula)	5^c^	2^c^	86^c^	52^c^	NA	NA
Seto et al^12^	2007	29	NA	0	NA	0	NA	24	83/33/33	23/0/0
Font et al^14^	2007	19	17^b^ (esophagitis)	NA	NA	6^b^	NA	NA	NA	NA
Fujita et al^13^	2005	23	30^a^ (leukocytopenia) 13^a^ (anemia)	NA	NA	NA	57	39	NA	NA
Ishida et al^38^ (JCOG9516)	2004	60^c^	33^c^ (leukocytopenia) 10^c^ (liver dysfunction)	NA	NA	3^c^	68^c^	15^c^	NA	NA
Crosby et al^15^	2004	27	12^b^ (oral mucositis) 10^b^ (leukocytopenia)	NA	NA	0	NA	NA	NA	NA
Kaneko et al^16^	2003	35	33^b^ (anemia) 30^b^ (leukocytopenia) 25^b^ (esophagitis)	0	9	6	NA	29	NA	NA
Nishimura et al^19^	2002	23	50 (leukocytopenia) 32 (dysphagia) 21 (anemia) 11 (thrombocytopenia)	NA	18	7	88	32	NA	NA
Itoh et al^17^	2001	35	NA	NA	NA	NA	68	17	83/25/25	26/7/0
Ohtsu et al^18^	1999	36	28^c^ (anemia) 24^c^ (leukocytopenia) 17^c^ (thrombocytopenia) 15^c^ (esophagitis)	NA	14	7^c^	81	25	NA	NA
Ohtsu et al^39^	1995	20^c^	45^c^ (leukocytopenia)	NA	20^c^	10^c^	85^c^	30^c^	NA	NA

Data in patients including ^a^Chemoradiotherapy (CRT) and CRT plus surgery group, ^b^T3/4 tumors, or ^c^T4/M1 lym tumors. cCR, clinical complete response; CR, complete response; LDCF, low‐dose cisplatin and 5‐fluorouracil; NA, data not available; SDCF, standard‐dose cisplatin and 5‐fluorouracil.

#### 3.2.2 Adverse effects, morbidity, and mortality

The most common early adverse effects associated with dCRT were hematotoxicities, including leukocytopenia, neutropenia, and thrombocytopenia (Table 2). In contrast, esophagitis, anorexia, oral mucositis, and esophageal dysphagia were common non‐hematological toxicities (Table 2). Fistula formation, including esophagotracheal (bronchial or pulmonary) and esophago‐aortic fistulas, was observed in 9%‐22%^16^, ^18^, ^19^, ^33^, ^34^, ^35^, ^36^, ^37^, ^39^, ^40^ of patients with cT4 esophageal cancer during or after dCRT. In the JCOG0303 trial,^36^ which included patients with cT4 and/or unresectable regional lymph node metastasis, grades 3‐4 fistula formation occurred in 32 of 140 patients (23%) during or after dCRT. Intercurrent deaths occurred (massive bleeding from an esophageal‐aortic fistula and pneumonia due to an esophageal‐pulmonary fistula) in two patients that were treated with a standard CF dose and 60 Gy radiation. One concurrent death was caused by massive bleeding as a result of an esophageal‐tracheal fistula. Chiarion‐Sileni et al^43^ reported that tracheo‐esophageal fistulas developed in 24% of patients with unresectable locally advanced ESCC treated with DCF, followed by carboplatin and radiotherapy. Nishimura et al^19^ studied 28 patients with T4 ESCC that underwent dCRT (60 Gy/CF). They reported worsening or development of esophageal fistulas in five (18%) patients and two (7%) treatment‐related deaths.

Incidence of late toxicities as a result of dCRT was 0%‐22%, although only five studies reported relevant data.^12^, ^16^, ^34^, ^36^, ^37^ In addition to fistula formation, the JCOG0303 study^36^ reported other common late toxicities, including dyspnea, dysphagia/esophagitis/odynophagia, and pneumonitis. Seto et al^12^ followed nine patients that survived more than 1 year from the start of dCRT; they reported no late toxicity‐related deaths, but they observed grade 2 pericardial effusion and radiation pneumonitis in four and two patients, respectively. An analysis of data from 14 studies^12^, ^14^, ^15^, ^16^, ^18^, ^19^, ^33^, ^34^, ^35^, ^36^, ^37^, ^38^, ^39^, ^40^ with relevant data indicated that the dCRT‐related mortality rate ranged from 0% to 10%. The main causes of dCRT‐related deaths were esophageal fistula with massive bleeding^16^, ^18^, ^19^ and pneumonitis.^14^

#### 3.2.3 Response to dCRT and patient survival

We found that patients with T4 tumors experienced a clinical complete response (cCR) of 0%‐39% and an overall response rate (both complete and partial responses) of 57%‐88% (Table 2).^16^, ^17^, ^18^, ^19^, ^33^, ^35^, ^36^, ^37^, ^38^, ^39^, ^40^ In contrast, the 1‐, 3‐, and 5‐year overall survival (OS) rates of patients with T4 esophageal cancer that received dCRT were 26%‐79%, 0%‐44%, and 0%‐14%, respectively.^12^, ^13^, ^14^, ^15^, ^16^, ^17^, ^18^, ^19^, ^33^, ^34^, ^35^, ^36^, ^37^, ^38^, ^40^ Seto et al^12^ examined prognosis according to the response to CRT; they reported that the 1‐, 3‐, and 5‐year survival rates of patients that experienced cCR and non‐cCR were 83%, 33%, 33%, and 23%, 0%, 0%, respectively. Itoh et al^17^ also reported that patients that achieved cCR had a significantly better prognosis than those with non‐cCR (1‐, 3‐, 5‐year OS rates: 83%, 25%, 25% vs 26%, 7%, 0%; *P *=* *0.0317). In a phase I/II study of DCF with concurrent radiation (60 Gy), Miyazaki et al^35^ reported that the CR rate and overall response rate were 48% and 86%, respectively, in patients with cT4 esophageal cancer. Accordingly, the prognosis of patients with T4 esophageal cancer that received dCRT depends on whether cCR can be achieved. However, patients that achieved cCR after dCRT sometimes developed disease recurrence. Therefore, careful follow up is necessary, even after achieving cCR. In addition, for recurrence or persistent disease after cCR, salvage surgery or palliation may be indicated, depending on the clinical situation and the patient's general condition.

### 3.3 Conversion surgery following induction treatments

#### 3.3.1 Regimen

As shown in Tables 1 and 3, 14 studies^12^, ^13^, ^20^, ^21^, ^22^, ^23^, ^24^, ^25^, ^26^, ^27^, ^28^, ^29^, ^41^, ^42^ have analyzed the outcome of patients with T4 esophageal cancer that underwent CS following induction treatments of CRT (often given with a radiation dose of 40‐50.4 Gy with the intention to explore the possibility of carrying out curative surgery later on) or chemotherapy. In all studies, the combination of CF with concurrent 36‐60 Gy irradiation was the most common regimen used as primary treatment.^12^, ^13^, ^20^, ^21^, ^22^, ^25^, ^26^, ^27^, ^28^, ^29^, ^41^, ^42^ All CRT in these series were carried out as a “planned” treatment before surgical resection; therefore, after induction treatment, the indication for CS was the relief of T4 invasion The interval between the completion of CRT and CS was 3‐8 weeks in all studies with available related data (Table 3).^13^, ^20^, ^21^, ^22^, ^24^, ^26^, ^27^, ^29^, ^42^ Alternatively, some more recent studies applied triplet chemotherapy regimens, including DCF, ACF,^20^, ^24^ and the combination of 5‐FU, adriamycin, and nedaplatin (FAN).^22^

**Table 3:** Summary of outcomes in conversion surgery group

Authors	N	Interval^a^ (weeks)	Combined resection rate (%)	Postoperative complications (%)	Mortality (%)	Resection rate^b^ (%)	Curative resection rate^b^ (%)	Clinical response rate (%)	pCR rate (%)		1/3/5‐year overall survival rate (%)	
									Main	All	Grade 3	Grade 0‐2
Yokota et al^20^ (COSMOS)	20	within 8	0	38 (recurrent nerve palsy) 24 (pleural effusion) 14 (lung infection)	0	42	40	NA	20	20	NA	NA
Ohira et al 41	40	NA	0	NA	NA	45	40	67	NA	NA	NA	NA
Akutsu et al^21^	28 (early responders) 12 (late responders)	3‐4	NA	NA	0 8	26	NA	100	22 17	NA NA	NA	NA
Shimoji et al^22^	43	4 (chemo) 6 (CRT)	0	63	13	70	61	54	14	14	NA	NA
Pimiento et al^23^	45	NA	0	total 52 22 (respiratory) 17 (DGE)	4	NA	96	NA	42	42	85/61/53	NA
Miyata et al^24^	98	3‐4	NA	NA	NA	58	47	78	16	16	NA	NA
Miyoshi et al^26^	42	4	NA	NA	NA	NA	NA	83	21	21	90/78/78	58/30/30
Seto et al^12^	59	NA	17 (respiratory tract) 10 (lung) 10 (pericardium)	NA	5	NA	NA	68	14	7	NA	NA
de Manzoni et al^25^	51	NA	NA	NA	10	78	39	20	NA	13	NA	NA
Fujita et al^13^	30	4‐6	0	total 87 50 (recurrent nerve palsy) 35 (respiratory) 23 (tracheal ischemia) 23 (pyothorax)	7	57	34	63	15	7	NA	NA
Noguchi et al^27^	41	4‐6	0	total 29 17 (anastomotic leak)	21	59	NA	59	17	17	100/75/25	20/0/0
Ikeda et al^28^	37	NA	0	NA	0	35	32	76	8	8	NA	NA
Yano et al^29^	45	4	NA	total 62 43 (respiratory) 25 (delirium) 21 (recurrent nerve palsy)	0	62	44	64	29	25	86/86/86	65/35/20
Van Raemdonck et al^42^	18	4‐8	0	11 (recurrent nerve palsy) 11 (surgical site infection) 11 (lymphatic fistula)	0	100	83	50	17	17	100/100/NA	53/32/NA

Chemo, chemotherapy; CR, complete response; CRT, chemoradiotherapy; DGE, delayed gastric emptying; NA, data not available; pCR, pathological complete response.

^a^Interval from the completion of chemoradiotherapy to the operation. ^b^Calculated with intention‐to‐treat analysis.

#### 3.3.2 Toxicity and mortality as a result of induction CRT or chemotherapy

Yano et al^29^ reported that the most common major toxicities (grade 3‐4) caused by CRT (40 Gy/CF) were leukocytopenia (49%), followed by gastrointestinal toxicities (47%). In that study, one patient (2%) died of a treatment‐related cause (pancytopenia). Ikeda et al^28^ reported that CRT (60 Gy/CF) caused grade 3 toxicity, particularly hematological reactions, in 13.5% (5/37) of patients (14% anemia and 14% leukocytopenia). They also observed one toxicity‐related death (sepsis). In addition, two patients developed esophagobronchial fistulas, two developed esophagovascular fistulas, and one developed an esophagomediastinal fistula. In the phase II study of chemoselection with DCF chemotherapy and subsequent CS (ie, the COSMOS trial),^20^ the major hematological toxicities (grades 3‐4) as a result of induction DCF chemotherapy were leukopenia (41.7%) and neutropenia (66.6%). Moreover, despite an antibiotic prophylaxis application, febrile neutropenia occurred in 11 (22.9%) patients. The most common non‐hematological adverse events, above grade 3, were anorexia (25.0%), diarrhea (10.4%), and nausea (4.2%). However, no grade 4 non‐hematological adverse event or treatment‐related death was observed during induction DCF. Two patients developed treatment‐related esophageal fistulas. In contrast, in that same trial, CRT was associated with several grade 3 hematological toxicities, including leukopenia (27.8%), neutropenia (5.6%), and anemia (11.1%). Moreover, grade 3 non‐hematological toxicities occurred, including esophagitis, dysphasia, anorexia, and nausea (n = 1 each). No esophageal fistula occurred with CRT. Several late complications occurred after CRT, including grade 1‐2 pneumonitis, grade 1 lung abscess, grade 3 esophagitis, and grade 3 anorexia. There was one treatment‐related death (respiratory bleeding) in a patient that received DCF chemotherapy followed by CRT (60 Gy).

#### 3.3.3 Resection and curative resection rates

Intention‐to‐treat (ITT) analysis showed that the rates of resection and curative resection (R0) for T4 diseases ranged from 26% to 100% and from 32% to 96%, respectively (Table 3).^13^, ^20^, ^21^, ^22^, ^23^, ^24^, ^25^, ^27^, ^28^, ^29^, ^41^, ^42^ Seto et al^12^ analyzed data for 59 patients with cT4 that underwent CS; they reported that 10 (17%), six (10%), and six (10%) patients underwent combined resections of the major respiratory tract, lung, or pericardium, respectively. However, no combination resection was used in the other studies (Table 3). Although Pimiento et al^23^ reported a curative resection rate of 96% after induction CRT, the most commonly invaded organ in that study was the pleura (75.6%), which was categorized as cT4a, but not cT4b, based on the UICC classification.

#### 3.3.4 Perioperative morbidity and mortality

Ranges of perioperative morbidity and mortality rates were 29%‐87% and 0%‐21%,^12^, ^13^, ^20^, ^21^, ^22^, ^23^, ^25^, ^27^, ^28^, ^29^, ^42^ respectively. Fujita et al^13^ analyzed patients with T4 tumors that underwent CS after CRT (36 Gy/CF); they reported an overall postoperative mortality rate of 8% (n = 2/26) and postoperative complications in 85% of patients (n = 22/26). The complications included 50% recurrent nerve palsy, 35% respiratory complications, 23% tracheal ischemia, and 23% pyothorax. Yano et al^29^ analyzed 45 patients that received CS after CRT (40 Gy/CF); they reported respiratory complications, delirium, and recurrent nerve palsy in 43%, 25%, and 21% of patients, respectively, with an overall morbidity rate of 62% (n = 28/45). Noguchi et al^27^ indicated a morbidity rate of 29% (7/24) among patients that received CS after CRT (40 Gy/CF). They found that anastomotic leakage was the most frequent complication (17%). Overall postoperative mortality rate after surgical resection was 21% (n = 5/24). Of these five postoperative deaths, two were related to postoperative complications involving anastomotic leaks, one died from postoperative pneumonia, one from liver failure, and one from catheter sepsis. In the COSMOS trial,^20^ no intraoperative complications were observed, but perioperative complications occurred, including recurrent laryngeal nerve palsy (38%), pleural effusion (24%), and lung infection (14%). Grade 3 severity rates were 5% for recurrent laryngeal nerve palsy, 5% for lung infections, 5% for wound infections, 5% for pulmonary fistulas, and 5% for dysphagia, but all of these complications were manageable. No grade 4 complications were observed; thus, there was no mortality and no serious complications related to surgery.

#### 3.3.5 Tumor response and survival

We found that 20%‐100% of patients with T4 esophageal cancer that received CS achieved a clinical response to induction CRT or chemotherapy (Table 3).^12^, ^13^, ^21^, ^22^, ^24^, ^25^, ^26^, ^27^, ^28^, ^29^, ^41^, ^42^ However, tumor examination showed that induction CRT or chemotherapy achieved pCR in 8%‐42% of cases only for the main tumor, and in 7%‐42% of cases only for all involved lesions.^12^, ^13^, ^21^, ^22^, ^23^, ^24^, ^25^, ^26^, ^27^, ^28^, ^29^, ^42^ The 1‐, 3‐, and 5‐year OS rates of T4 patients that underwent CS were 24%‐100%, 5%‐50%, and 0%‐51%, respectively.^12^, ^13^, ^20^, ^21^, ^22^, ^23^, ^24^, ^25^, ^26^, ^27^, ^28^, ^29^, ^41^, ^42^ Among the five studies^23^, ^26^, ^27^, ^29^, ^42^ that classified prognosis according to the pathological response to CRT, 1‐, 3‐, and 5‐year OS rates were 85%‐100%, 61%‐100%, and 25%‐86%, respectively, for grade 3 tumors, and 20‐65%, 0‐35%, and 0%‐30% for grade 0‐2 tumors (Table 3).

Miyata et al^24^ analyzed 98 patients that underwent CRT or triplet chemotherapy plus CRT, with or without subsequent CS; they found that patients that underwent CS had significantly more favorable 3‐ and 5‐year OS rates (48% and 40%, respectively) compared to patients that did not receive CS (7% and 4%, respectively). This trend was also identified in patients that showed a good response to induction treatments and those that showed a poor response using separately analyzed survival data (data not shown). Seto et al^12^ reported that the 1‐ and 3‐year OS rates of 59 patients with cT4 ESCC who underwent neoadjuvant CRT plus esophagectomy were 67.8% and 37.9%, respectively. The 1‐ and 3‐year OS rates were 77.8% and 45.1%, respectively, for R0 resections and 38.5% and 0%, respectively, for palliative resections (R1/2). The prognosis of patients that underwent tracheal resections was poor, even after a R0 resection. de Manzoni et al^25^ analyzed the survival of patients with esophageal cancer according to the infiltrated organs detected on pretreatment staging; they reported that curative resections were possible after CRT (50‐60 Gy/CF) when tumors invaded the aorta, but no long‐term survivors were observed when tumors had invaded other organs. Among patients with invasions of the aorta, airway, and other organs, the 3‐year survival times were 31.3, 4.5, and 0 months, respectively.^25^ Furthermore, median survival times were 22.3 and 9 months for patients with R0 and R1/2 resections, respectively (*P *<* *0.001). The recurrence pattern after a CS for cT4 esophageal cancer was only described in one study by Yano et al^29^ They reported that, among 27 patients, 17 (63%) experienced recurrence after a curative resection; among these 17 recurrences, eight were local, six were distant, two were local plus distant, and one displayed an unknown.

### 3.4 Triplet chemotherapy as an initial induction treatment

The standard regimen for induction treatment in locally advanced T4 esophageal cancer is concurrent CRT with CF. The CF regimen has not changed in decades, but it is possible that a stronger regimen might improve outcomes. In 2007, a novel regimen of DCF achieved a significant antitumor effect and improved the outcome of patients with head and neck cancer.^44^ DCF was also expected to be effective for ESCC because of its histological similarity to head and neck cancer. Indeed, DCF had a strong antitumor effect for ESCC, and it is currently being used as a first‐line chemotherapy regimen for ESCC. DCF even achieved local tumor control comparable to that achieved with CRT; thus, several studies^30^, ^31^, ^32^ used DCF as an initial induction treatment for T4 ESCC and confirmed its clinical utility. A recent phase II study (COSMOS trial)^20^ investigated the efficacy of induction DCF chemotherapy. That study aimed to test downstaging the tumor and, subsequently, converting to surgery as a multidisciplinary strategy for treating cT4 ESCC. In that trial, the first‐line chemotherapy regimen consisted of three courses of DCF. When resectability was achieved after the third course of DCF, CS was carried out. When resectability was not achieved by the middle evaluation of CRT, dCRT was given. That study reported that CS was carried out in 41.7% of patients, and an R0 resection was confirmed in 39.6% of patients. A point estimate of the 1‐year survival rate was 67.7%, and the 80% confidence interval had a lower limit of 59.5%. Because this lower limit was higher than the 50% threshold, this first prospective trial showed a statistically positive effect. In addition, the 1‐year survival rate in that study was higher than that found in the standard‐dose CF‐RT arm in the JCOG0303 trial.^36^ This finding indicated that DCF chemotherapy was a sufficiently powerful induction treatment for cT4 ESCC.

Miyata et al^24^ investigated the clinical utility of initial induction triplet chemotherapy with either a DCF or an ACF regimen, with or without a second‐line induction CRT, for treating cT4 ESCC. In that study, induction DCF chemotherapy reduced esophageal perforations and increased overall resectability in patients with T4 ESCC, which led to a better survival rate than that achieved with CRT alone. Makino et al,^30^ from the same institute, carried out a propensity score‐matched analysis. They compared 50 patients with cT4 ESCC that underwent an initial DCF induction therapy to another 50 patients that underwent induction radiotherapy concurrent with a CF regimen (CRT); they reported that the initial induction DCF chemotherapy achieved up to 64% of the clinical response rate which was nearly comparable to the 72.0% achieved with induction CRT. Compared to the CRT group, the DCF group had significantly higher overall resectability (78.0% vs 48.0%, *P *=* *0.0017) and survival (5‐year cancer‐specific survival: 42.1% vs 22.2%, *P *=* *0.0146). Considering that local recurrence after curative surgery tended to be lower in the DCF group than in the CRT group, DCF chemotherapy appeared to control local disease sufficiently, with or without subsequent CRT. Another potential benefit of giving induction DCF chemotherapy for T4 ESCC is to control micrometastasis; this application was supported by the finding that survival superiority with DCF was observed only for the node‐positive (cN1‐3) population. Shimoji et al^22^ conducted a prospective study on a cohort that received FAN induction triplet chemotherapy (n = 17) or CRT (n = 26) each treatment followed, when feasible, by esophagectomy. They also reported that satisfactory survival could be achieved when R0 resection was carried out after induction treatment in T4 ESCC; however, a secondary radical esophagectomy was associated with a higher risk of in‐hospital mortality.

Satake et al^33^ conducted a multicenter phase I/II study on induction DCF chemotherapy followed by CRT in patients with unresectable, locally advanced ESCC. In that trial, DCF induction chemotherapy showed promising efficacy with a median progression‐free survival of 12 months and a 3‐year survival rate of 40.4%. However, 39.4% of the 33 patients with ESCC that involved cT4 and/or M1 lym achieved a CR; this CR rate was less than expected. A post‐JCOG0303 trial^36^ was recently started to test a trimodality combination therapy with induction DCF compared to dCRT for locally advanced unresectable (cT4) ESCC of the thoracic esophagus (TRIANgLE; JCOG1510). The aim of this new phase III JCOG study is to confirm that DCF chemotherapy followed by radical surgery or dCRT shows superiority in OS over the standard dCRT for patients with cT4 ESCC of the thoracic esophagus. The primary endpoint of the trial is OS. Secondary endpoints include progression‐free survival, (complete) response rate, adverse events of DCF or CRT, late‐onset adverse events, and perioperative complications. A total of 230 patients will be recruited from 47 Japanese institutions.

## 4 SUMMARY AND PERSPECTIVES

**Figure 1:**
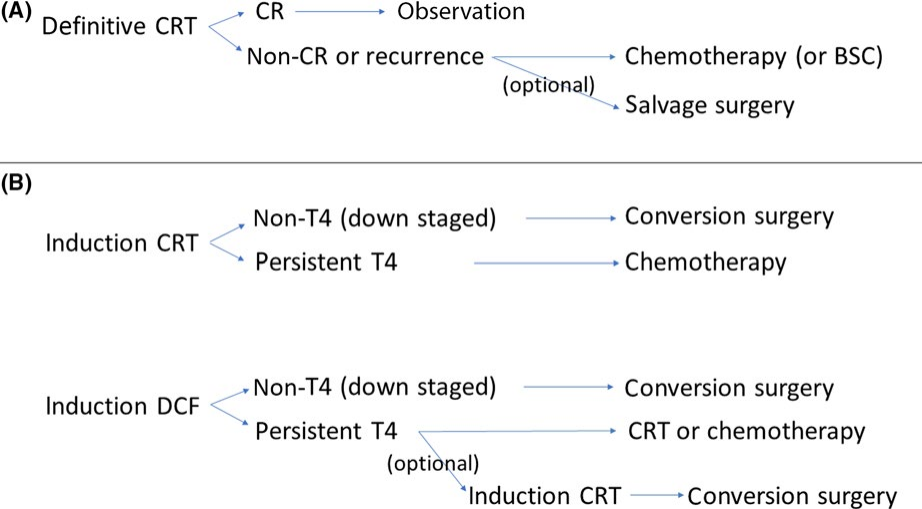
Possible algorism of a treatment strategy for cT4 esophageal cancer. Different treatment strategies, including (A) definitive chemoradiation (CRT), potentially followed by salvage surgery, in the absence of a complete response (CR); or (B) induction treatments potentially followed by conversion surgery. BSC, best supportive care; DCF, docetaxel, cisplatin, and 5‐fluorouracil

A possible algorism of treatment for cT4 esophageal cancer is summarized in Figure 1. In the case of dCRT, patient prognosis depends on whether or not cCR can be achieved. However, it is often difficult to determine a treatment strategy after achieving cCR with dCRT. It is also clinically difficult to make a diagnosis of CR based on endoscopic biopsies, which sometimes give false‐negative results, or imaging tools, due to CRT‐induced inflammation, fibrosis, or edema. In contrast, it remains controversial whether surgery should play a role in a treatment modality carried out after achieving CR with dCRT. Two randomized trials^45^, ^46^ have compared preoperative dCRT, followed by surgery, versus dCRT alone to assess the role of surgery in T3 and/or T4 diseases. They found that adding surgery to dCRT provided no survival benefit. Furthermore, significantly higher operative mortality rates and major morbidities, including anastomotic leaks and pulmonary complications, were reported in both trials. These findings were presumably due to the adverse effects of CRT, including radiation‐induced fibrosis, which affected thoracic tissue and patient performance status. Meanwhile, as patients that achieved cCR after dCRT sometimes developed disease recurrence, careful follow up is necessary even after achieving cCR. In addition, for recurrence or persistent disease (non‐CR) after cCR, salvage surgery (optional) or palliation including chemotherapy may be indicated, depending on the clinical situation and the patient's general condition (Figure 1A). However, when curative resection is considered possible after induction CRT or DCF, CS might be scheduled. When the tumor remains unresectable (persistent T4), chemotherapy or CRT might subsequently be given, depending on the type of initial induction treatment. In cases with persistent T4 tumors after an initial induction with DCF, a second‐line induction CRT might be indicated to pursue any chance of carrying out CS as an optional treatment strategy; this latter option is practiced in our institute (Figure 1B).

Older patients are often excluded, or at least underrepresented, in clinical trials. Thus, it is reasonable to question whether the results are generally transferable to the older population. Although it is true that some older patients are not suitable for intensive multimodality treatment, age alone should not be taken as the decisive factor in making treatment decisions in T4 esophageal cancer. In fact, according to a recent analysis by Pultrum et al.,^47^ older age did not significantly influence the overall outcome or the complication rate in patients treated with extended esophagectomies, However, the presence of comorbidity had a significant impact on survival. Thus, it might be more appropriate to base treatment decisions on comorbidity and/or performance status, rather than chronological age alone.^48^ Although we proposed a possible treatment algorithm for cT4 esophageal cancer (Figure 1), the tolerance for each treatment should first be evaluated, considering comorbidity, performance status, and general condition, in addition to the patient's age. Radiation alone or palliation might be indicated for older patients at high risk; alternatively, a potentially curative treatment strategy might be considered for carefully selected older patients without severe comorbidity.

This review has shown that CS appeared to be superior to dCRT for treating T4 esophageal cancer with respect to local control and short‐term prognosis despite the relatively high association with perioperative morbidities. However, although the fistula formation rate was relatively high in dCRT, a CR to CRT might lead to a better prognosis. When more powerful chemotherapy, such as a DCF regimen, is tolerable concurrent with definitive radiation, this is the most promising option for treating T4 esophageal cancer. Also, as an initial induction therapy, triplet chemotherapy, including a DCF regimen, can yield both significant local control and systemic control, which enables the application of CS for T4 esophageal cancer, without preoperative radiation. DCF chemotherapy can also be used for chemoselection, followed by CS or dCRT, as a multidisciplinary treatment strategy. In addition, a number of clinical trials are currently testing immune‐checkpoint inhibitors with/without chemotherapy or radiation. These treatments might become viable treatment options for T4 esophageal cancer in the near future. Randomized controlled trials that include a large population are needed to define a standard treatment for T4 esophageal cancer.

## DISCLOSURE

Authors declare no conflicts of interest for this article.

## References

[1] Makino T , Doki Y . Treatment of T4 esophageal cancer. Definitive chemo‐radiotherapy vs chemo‐radiotherapy followed by surgery. Ann Thorac Cardiovasc Surg. 2011;17:221–8. 21697781 10.5761/atcs.ra.11.01676

[2] Gamliel Z , Krasna MJ . Multimodality treatment of esophageal cancer. Surg Clin North Am. 2005;85:621–30. 15927656 10.1016/j.suc.2005.01.011

[3] Makino T , Yamasaki M , Tanaka K , et al. Importance of positron emission tomography for assessing the response of primary and metastatic lesions to induction treatments in T4 esophageal cancer. Surgery. 2017;162:836–45. 28711321 10.1016/j.surg.2017.06.007

[4] Shimada H , Okazumi S , Matsubara H , et al. Impact of the number and extent of positive lymph nodes in 200 patients with thoracic esophageal squamous cell carcinoma after three‐field lymph node dissection. World J Surg. 2006;30:1441–9. 16871357 10.1007/s00268-005-0462-6

[5] Shimada H , Kitabayashi H , Nabeya Y , et al. Treatment response and prognosis of patients after recurrence of esophageal cancer. Surgery. 2003;133:24–31. 12563234 10.1067/msy.2003.31

[6] Ichiyoshi Y , Kawahara H , Taga S , et al. Indications and operative techniques for combined aortoesophageal resection. Jpn J Thorac Cardiovasc Surg. 1999;47:318–24. 10481389 10.1007/BF03218018

[7] Shenfine J , McNamee P , Steen N , et al. A pragmatic randomised controlled trial of the cost‐effectiveness of palliative therapies for patients with inoperable oesophageal cancer. Health Technol Assess. 2005;9:iii, 1–121. 10.3310/hta9050 15717937

[8] Akutsu Y , Matsubara H . Chemoradiotherapy and surgery for T4 esophageal cancer in Japan. Surg Today. 2015;45:1360–5. 25583206 10.1007/s00595-015-1116-4

[9] Ancona E , Ruol A , Castoro C , et al. First‐line chemotherapy improves the resection rate and long‐term survival of locally advanced (T4, any N, M0) squamous cell carcinoma of the thoracic esophagus: final report on 163 consecutive patients with 5‐year follow‐up. Ann Surg. 1997;226:714–723. 9409570 10.1097/00000658-199712000-00008 PMC1191144

[10] John MJ , Flam MS , Mowry PA , et al. Radiotherapy alone and chemoradiation for nonmetastatic esophageal carcinoma. A critical review of chemoradiation. Cancer. 1989;63:2397–403. 2720585 10.1002/1097-0142(19890615)63:12<2397::aid-cncr2820631204>3.0.co;2-s

[11] Tsujinaka T , Shiozaki H , Yamamoto M , et al. Role of preoperative chemoradiation in the management of upper third thoracic esophageal squamous cell carcinoma. Am J Surg. 1999;177:503–506; discussion 507. 10414703 10.1016/s0002-9610(99)00103-8

[12] Seto Y , Chin K , Gomi K , et al. Treatment of thoracic esophageal carcinoma invading adjacent structures. Cancer Sci. 2007;98:937–42. 17441965 10.1111/j.1349-7006.2007.00479.x PMC11159274

[13] Fujita H , Sueyoshi S , Tanaka T , et al. Esophagectomy: is it necessary after chemoradiotherapy for a locally advanced T4 esophageal cancer? Prospective nonrandomized trial comparing chemoradiotherapy with surgery versus without surgery. World J Surg. 2005;29:25–30. 15599735 10.1007/s00268-004-7590-2

[14] Font A , Arellano A , Fernandez‐Llamazares J , et al. Weekly docetaxel with concomitant radiotherapy in patients with inoperable oesophageal cancer. Clin Transl Oncol. 2007;9:177–82. 17403629 10.1007/s12094-007-0032-5

[15] Crosby TD , Brewster AE , Borley A , et al. Definitive chemoradiation in patients with inoperable oesophageal carcinoma. Br J Cancer. 2004;90:70–5. 14710209 10.1038/sj.bjc.6601461 PMC2395332

[16] Kaneko K , Ito H , Konishi K , et al. Definitive chemoradiotherapy for patients with malignant stricture due to T3 or T4 squamous cell carcinoma of the oesophagus. Br J Cancer. 2003;88:18–24. 12556953 10.1038/sj.bjc.6600684 PMC2376792

[17] Itoh Y , Fuwa N , Matsumoto A , et al. Outcomes of radiotherapy for inoperable locally advanced (T4) esophageal cancer‐retrospective analysis. Radiat Med. 2001;19:231–5. 11724253

[18] Ohtsu A , Boku N , Muro K , et al. Definitive chemoradiotherapy for T4 and/or M1 lymph node squamous cell carcinoma of the esophagus. J Clin Oncol. 1999;17:2915–21. 10561371 10.1200/JCO.1999.17.9.2915

[19] Nishimura Y , Suzuki M , Nakamatsu K , et al. Prospective trial of concurrent chemoradiotherapy with protracted infusion of 5‐fluorouracil and cisplatin for T4 esophageal cancer with or without fistula. Int J Radiat Oncol Biol Phys. 2002;53:134–9. 12007951 10.1016/s0360-3016(01)02813-9

[20] Yokota T , Kato K , Hamamoto Y , et al. Phase II study of chemoselection with docetaxel plus cisplatin and 5‐fluorouracil induction chemotherapy and subsequent conversion surgery for locally advanced unresectable oesophageal cancer. Br J Cancer. 2016;115:1328–34. 27811857 10.1038/bjc.2016.350 PMC5129815

[21] Akutsu Y , Kono T , Uesato M , et al. Is the outcome of a salvage surgery for T4 thoracic esophageal squamous cell carcinoma really poor? World J Surg. 2014;38:2891–7. 24952078 10.1007/s00268-014-2668-y

[22] Shimoji H , Karimata H , Nagahama M , Nishimaki T . Induction chemotherapy or chemoradiotherapy followed by radical esophagectomy for T4 esophageal cancer: results of a prospective cohort study. World J Surg. 2013;37:2180–8. 23649529 10.1007/s00268-013-2074-x

[23] Pimiento JM , Weber J , Hoffe SE , et al. Outcomes associated with surgery for T4 esophageal cancer. Ann Surg Oncol. 2013;20:2706–12. 23504118 10.1245/s10434-013-2885-x

[24] Miyata H , Yamasaki M , Kurokawa Y , et al. Clinical relevance of induction triplet chemotherapy for esophageal cancer invading adjacent organs. J Surg Oncol. 2012;106:441–7. 22371189 10.1002/jso.23081

[25] de Manzoni G , Pedrazzani C , Pasini F , et al. Chemoradiotherapy followed by surgery for squamous cell carcinoma of the thoracic esophagus with clinical evidence of adjacent organ invasion. J Surg Oncol. 2007;95:261–6. 17323341 10.1002/jso.20640

[26] Miyoshi N , Yano M , Takachi K , et al. Myelotoxicity of preoperative chemoradiotherapy is a significant determinant of poor prognosis in patients with T4 esophageal cancer. J Surg Oncol. 2009;99:302–6. 19170110 10.1002/jso.21235

[27] Noguchi T , Moriyama H , Wada S , et al. Resection surgery with neoadjuvant chemoradiotherapy improves outcomes of patients with T4 esophageal carcinoma. Dis Esophagus. 2003;16:94–8. 12823205 10.1046/j.1442-2050.2003.00304.x

[28] Ikeda K , Ishida K , Sato N , et al. Chemoradiotherapy followed by surgery for thoracic esophageal cancer potentially or actually involving adjacent organs. Dis Esophagus. 2001;14:197–201. 11869319 10.1046/j.1442-2050.2001.00184.x

[29] Yano M , Tsujinaka T , Shiozaki H , et al. Concurrent chemotherapy (5‐fluorouracil and cisplatin) and radiation therapy followed by surgery for T4 squamous cell carcinoma of the esophagus. J Surg Oncol. 1999;70:25–32. 9989417 10.1002/(sici)1096-9098(199901)70:1<25::aid-jso5>3.0.co;2-m

[30] Makino T , Yamasaki M , Miyazaki Y , et al. Utility of initial induction chemotherapy with 5‐fluorouracil, cisplatin, and docetaxel (DCF) for T4 esophageal cancer: a propensity score‐matched analysis. Dis Esophagus 2018;31:10.1093/dote/dox130. 29190316

[31] Yamasaki M , Yasuda T , Yano M , et al. Multicenter randomized phase II study of cisplatin and fluorouracil plus docetaxel (DCF) compared with cisplatin and fluorouracil plus Adriamycin (ACF) as preoperative chemotherapy for resectable esophageal squamous cell carcinoma (OGSG1003). Ann Oncol. 2017;28:116–20. 27687307 10.1093/annonc/mdw439

[32] Shiraishi O , Yamasaki M , Makino T , et al. Feasibility of preoperative chemotherapy with docetaxel, cisplatin, and 5‐fluorouracil versus adriamycin, cisplatin, and 5‐fluorouracil for resectable advanced esophageal cancer. Oncology. 2017;92:101–8. 27907921 10.1159/000452765

[33] Satake H , Tahara M , Mochizuki S , et al. A prospective, multicenter phase I/II study of induction chemotherapy with docetaxel, cisplatin and fluorouracil (DCF) followed by chemoradiotherapy in patients with unresectable locally advanced esophageal carcinoma. Cancer Chemother Pharmacol. 2016;78:91–9. 27193097 10.1007/s00280-016-3062-2 PMC4921115

[34] Jingu K , Umezawa R , Matsushita H , et al. Chemoradiotherapy for T4 and/or M1 lymph node esophageal cancer: experience since 2000 at a high‐volume center in Japan. Int J Clin Oncol. 2016;21:276–82. 26324841 10.1007/s10147-015-0896-2

[35] Miyazaki T , Sohda M , Tanaka N , et al. Phase I/II study of docetaxel, cisplatin, and 5‐fluorouracil combination chemoradiotherapy in patients with advanced esophageal cancer. Cancer Chemother Pharmacol. 2015;75:449–55. 25544126 10.1007/s00280-014-2659-6

[36] Shinoda M , Ando N , Kato K , et al. Randomized study of low‐dose versus standard‐dose chemoradiotherapy for unresectable esophageal squamous cell carcinoma (JCOG0303). Cancer Sci. 2015;106:407–12. 25640628 10.1111/cas.12622 PMC4409884

[37] Higuchi K , Komori S , Tanabe S , et al. Definitive chemoradiation therapy with docetaxel, cisplatin, and 5‐fluorouracil (DCF‐R) in advanced esophageal cancer: a phase 2 trial (KDOG 0501‐P2). Int J Radiat Oncol Biol Phys. 2014;89:872–9. 24867539 10.1016/j.ijrobp.2014.03.030

[38] Ishida K , Ando N , Yamamoto S , et al. Phase II study of cisplatin and 5‐fluorouracil with concurrent radiotherapy in advanced squamous cell carcinoma of the esophagus: a Japan Esophageal Oncology Group (JEOG)/Japan Clinical Oncology Group trial (JCOG9516). Jpn J Clin Oncol. 2004;34:615–9. 15591460 10.1093/jjco/hyh107

[39] Ohtsu A , Yoshida S , Boku N , et al. Concurrent chemotherapy and radiation therapy for locally advanced carcinoma of the esophagus. Jpn J Clin Oncol. 1995;25:261–6. 8523823

[40] Li M , Zhao F , Zhang X , et al. Involved‐field irradiation in definitive chemoradiotherapy for T4 squamous cell carcinoma of the esophagus. Curr Oncol. 2016;23:e131–7. 27122981 10.3747/co.23.2846 PMC4835014

[41] Ohira M , Kubo N , Masuda G , et al. Glasgow prognostic score as a prognostic clinical marker in T4 esophageal squamous cell carcinoma. Anticancer Res. 2015;35:4897–901. 26254385

[42] Van Raemdonck D , Van Cutsem E , Menten J , et al. Induction therapy for clinical T4 oesophageal carcinoma; a plea for continued surgical exploration. Eur J Cardiothorac Surg. 1997;11:828–37. 9196296 10.1016/s1010-7940(97)01194-9

[43] Chiarion‐Sileni V , Corti L , Ruol A , et al. Phase II trial of docetaxel, cisplatin and fluorouracil followed by carboplatin and radiotherapy in locally advanced oesophageal cancer. Br J Cancer. 2007;96:432–8. 17245338 10.1038/sj.bjc.6603585 PMC2360020

[44] Posner MR , Hershock DM , Blajman CR , et al. Cisplatin and fluorouracil alone or with docetaxel in head and neck cancer. N Engl J Med. 2007;357:1705–15. 17960013 10.1056/NEJMoa070956

[45] Stahl M , Stuschke M , Lehmann N , et al. Chemoradiation with and without surgery in patients with locally advanced squamous cell carcinoma of the esophagus. J Clin Oncol. 2005;23:2310–7. 15800321 10.1200/JCO.2005.00.034

[46] Bedenne L , Michel P , Bouche O , et al. Chemoradiation followed by surgery compared with chemoradiation alone in squamous cancer of the esophagus: FFCD 9102. J Clin Oncol. 2007;25:1160–8. 17401004 10.1200/JCO.2005.04.7118

[47] Pultrum BB , Bosch DJ , Nijsten MW , et al. Extended esophagectomy in elderly patients with esophageal cancer: minor effect of age alone in determining the postoperative course and survival. Ann Surg Oncol. 2010;17:1572–80. 20180031 10.1245/s10434-010-0966-7 PMC2868167

[48] Walter F , Bockle D , Schmidt‐Hegemann NS , et al. Clinical outcome of elderly patients (>/= 70 years) with esophageal cancer undergoing definitive or neoadjuvant radio(chemo)therapy: a retrospective single center analysis. Radiat Oncol. 2018;13:93. 29769143 10.1186/s13014-018-1044-8 PMC5956563

