# Supplementary figures and images for: Treatment and clinical outcome of clinical T4 esophageal cancer: A systematic review
Source: Ann Gastroenterol Surg. 2018 Dec 13;3(2):169–80. doi: 10.1002/ags3.12222 (PMC6422802; doi:10.1002/ags3.12222)

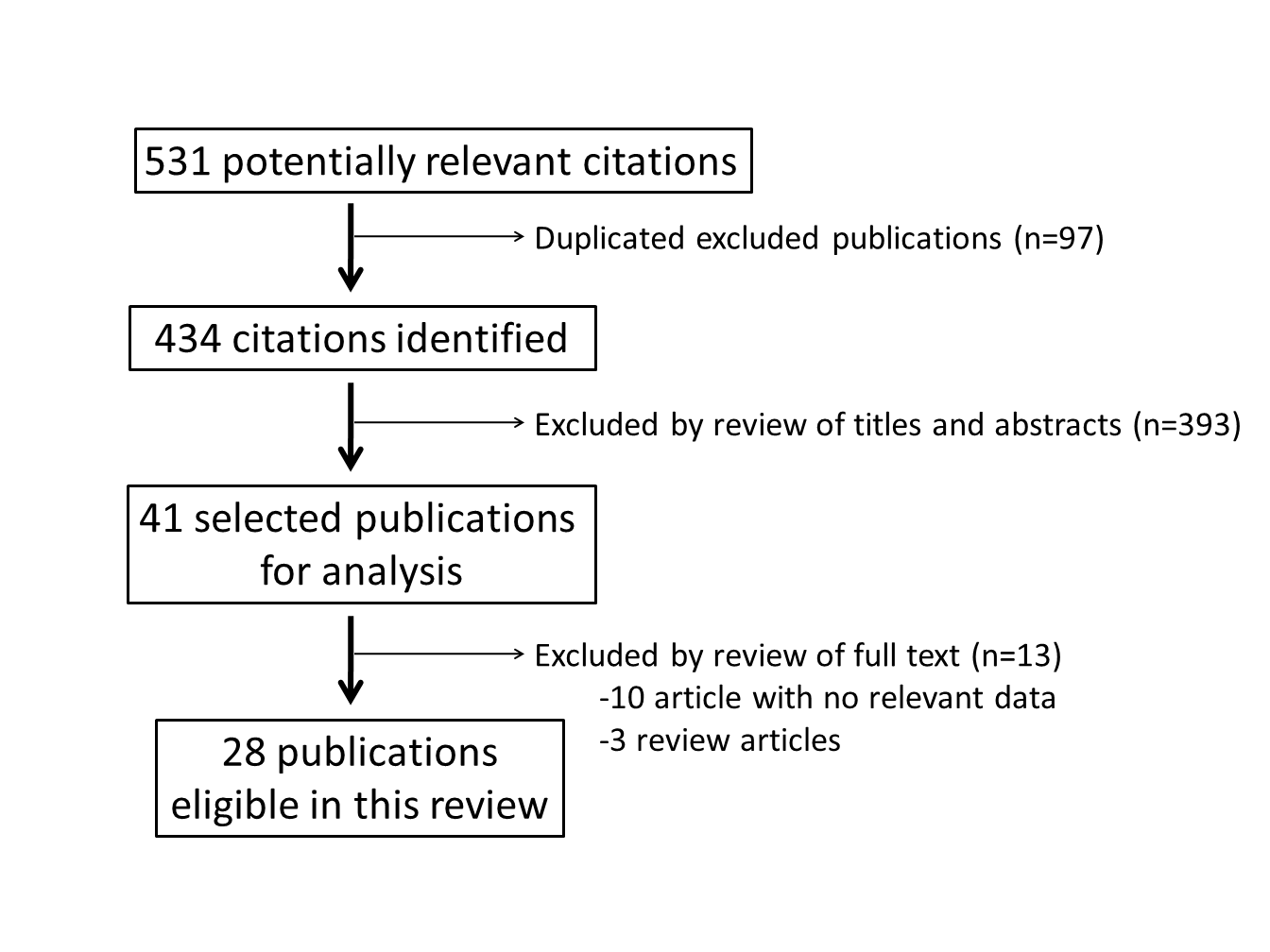

Supplement: Supplementary file 1 [file AGS3-3-169-s001.tif]
